# Supplementary material for: Mechanisms governing the pioneering and redistribution capabilities of the non-classical pioneer PU.1
Source: Nat Commun. 2020 Jan 21;11:402. doi: 10.1038/s41467-019-13960-2 (PMC6972792; doi:10.1038/s41467-019-13960-2)
Supplement: Supplementary file 7 — Source data [file 41467_2019_13960_MOESM7_ESM.zip › Source_Data/FigureS2/FigureS2B_MotifScanOutput/KmeansCluster4/homerResults/motif16.similar.html]

motif16

## Information for motif16

A
C
T
G
A
C
T
G
C
T
G
A
A
C
T
G
A
C
T
G
C
G
T
A
C
T
A
G
A
C
T
G
C
G
T
A
  
Reverse Opposite:  

A
C
G
T
A
G
T
C
A
G
T
C
A
C
G
T
A
G
T
C
A
G
T
C
A
G
C
T
A
G
T
C
A
G
T
C
  

|  |  |
| --- | --- |
| p-value: | 1e-56 |
| log p-value: | -1.291e+02 |
| Information Content per bp: | 1.959 |
| Number of Target Sequences with motif | 364.0 |
| Percentage of Target Sequences with motif | 8.06% |
| Number of Background Sequences with motif | 1211.5 |
| Percentage of Background Sequences with motif | 2.90% |
| Average Position of motif in Targets | 100.5 +/- 48.2bp |
| Average Position of motif in Background | 96.9 +/- 95.2bp |
| Strand Bias (log2 ratio + to - strand density) | 0.4 |
| Multiplicity (# of sites on avg that occur together) | 1.46 |
| Motif File: | file (matrix) reverse opposite |

### Similar de novo motifs found

|  |  |  |  |  |  |  |  |
| --- | --- | --- | --- | --- | --- | --- | --- |
| Rank | Match Score | Redundant Motif | P-value | log P-value | % of Targets | % of Background | Motif file |
| 1 | 0.760 | A G T C A G T C G C A T G A T C T G A C A G C T A G T C A G T C G A C T A G T C | 1e-49 | -112.953296 | 7.51% | 2.80% | motif file (matrix) |
| 2 | 0.939 | A C T G A C T G C G T A A C T G A C T G C G T A A C T G A C T G | 1e-19 | -43.956834 | 6.87% | 3.82% | motif file (matrix) |
| 3 | 0.875 | A C T G C G T A A C T G A C T G C G T A A C T G A C T G | 1e-14 | -33.890872 | 12.20% | 8.53% | motif file (matrix) |
